# The Herbal Bitter Drug *Gentiana lutea* Modulates Lipid Synthesis in Human Keratinocytes In Vitro and In Vivo

**DOI:** 10.3390/ijms18081814

**Published:** 2017-08-22

**Authors:** Ute Wölfle, Birgit Haarhaus, Jasmin Seiwerth, Anja Cawelius, Kay Schwabe, Karl-Werner Quirin, Christoph M. Schempp

**Affiliations:** 1 Department of Dermatology, Medical Center, Faculty of Medicine, University of Freiburg, 79085 Breisgau, Germany; birgit.haarhaus@uniklinik-freiburg.de (B.H.); Jasmin.Seiwerth@t-online.de (J.S.); Christoph.schempp@uniklinik-freiburg.de (C.M.S.); 2 Flavex Naturextrakte GmbH, 66780 Rehlingen, Germany; ac@flavex.com (A.C.); wq@flavex.com (K.-W.Q.); 3 BSI-Beauty Science Intelligence GmbH, 30855 Langenhagen, Germany; K.Schwabe@bsi-cosmetics.de

**Keywords:** keratinocytes, *Gentiana lutea*, lipid synthesis, p38 MAPK, PPARγ, ceramide synthase 3

## Abstract

*Gentiana lutea* is a herbal bitter drug that is used to enhance gastrointestinal motility and secretion. Recently we have shown that amarogentin, a characteristic bitter compound of *Gentiana lutea* extract (GE), binds to the bitter taste receptors TAS2R1 and TAS2R38 in human keratinocytes, and stimulates the synthesis of epidermal barrier proteins. Here, we wondered if GE also modulates lipid synthesis in human keratinocytes. To address this issue, human primary keratinocytes were incubated for 6 days with GE. Nile Red labeling revealed that GE significantly increased lipid synthesis in keratinocytes. Similarly, gas chromatography with flame ionization detector indicated that GE increases the amount of triglycerides in keratinocytes. GE induced the expression of epidermal ceramide synthase 3, but not sphingomyelinase. Lipid synthesis, as well as ceramide synthase 3 expression, could be specifically blocked by inhibitors of the p38 MAPK and PPARγ signaling pathway. To assess if GE also modulates lipid synthesis in vivo, we performed a proof of concept half side comparison on the volar forearms of 33 volunteers. In comparison to placebo, GE significantly increased the lipid content of the treated skin areas, as measured with a sebumeter. Thus, GE enhances lipid synthesis in human keratinocytes that is essential for building an intact epidermal barrier. Therefore, GE might be used to improve skin disorders with an impaired epidermal barrier, e.g., very dry skin and atopic eczema.

## 1. Introduction

The stratum corneum consists of terminally differentiated keratinocytes (corneocytes) that are surrounded by an extracellular lipid bilayer and bound water. Lipids for this bilayer are secreted from lipid droplets, which are intracellular organelles that are specialized in assembling, storing and then supplying lipids [1]. Although sebocytes are specialized in lipid synthesis and storage, almost every cell has the capacity to store and accumulate lipids in lipid droplets [2]. In keratinocytes, lipid droplets are generated when keratinocytes differentiate and reach their maximal number in the stratum granulosum, where also, the highest level of extracellular calcium exists [3].

There are various lipids that contribute to an optimal epidermal barrier function, of which ceramides, the backbone structure of all sphingolipids, are particular important. They represent nearly 50% of the lipid mass in the stratum corneum [4,5]. A decrease in the amount of ceramides occurs in multiple skin disorders with barrier abnormalities, for example, atopic dermatitis and psoriasis [6,7]. Altered expression of key enzymes that are involved in the ceramide metabolism in keratinocytes might account for this, e.g., ceramide synthase 3 (CerS3) and acidic sphingomyelinase. CerS3 is a crucial enzyme for the synthesis of very long-chain ceramides in the epidermis. These ceramides are important for maintaining epidermal lipid homeostasis and terminal differentiation [8]. Acidic sphingomyelinases catalyze the hydrolysis of sphingomyelin (ceramide phosphorylcholine) into ceramide and phosphorylcholine, and have also been related to cellular processes, such as apoptosis, cell proliferation, cell differentiation, and membrane fusion/fission [9]. Furthermore, during aging, stratum corneum ceramides decrease by approximately 10–15% per decade after the age of 20 years [10]. Aging also influences the composition and amount of free fatty acids [11]. A deficiency in fatty acids (e.g., by delayed fatty acid synthesis) results in abnormalities in the stratum corneum structure and delayed recovery of the permeability barrier.

**Figure 1:**
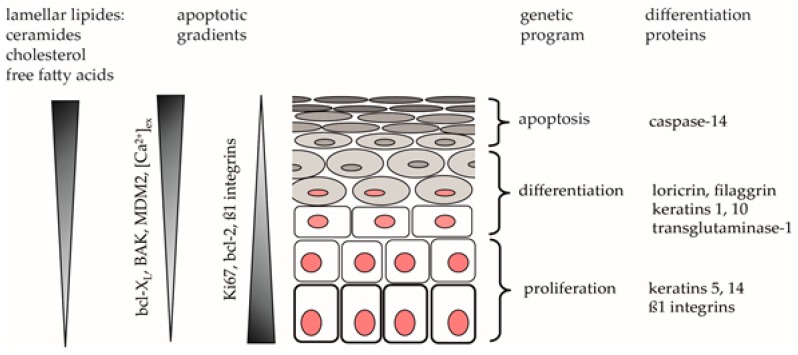
Illustration of the differentiation process in keratinocytes. The triangles indicate increasing or decreasing concentrations of the respective molecules within the epidermis. Bcl-2—B-cell lymphoma 2; BAK—Bcl-2 antagonist/killer1; MDM2—mouse double minute 2 homolog; Ca^2+^—calcium. The graphic was adapted from [12].

A complete skin barrier requires not only an intact lipid layer but also terminal differentiation proteins (see Figure 1).

Generally, differentiation of keratinocytes is induced by an increase of the intracellular calcium concentration. Recently, we could show that amarogentin, a characteristic bitter compound of *Gentiana lutea*, induces calcium influx in keratinocytes and promotes keratinocyte differentiation by inducing keratin 10, involucrin, and transglutaminase expression [13]. Additionally, we demonstrated that human keratinocytes express the bitter taste receptors TAS2R1 and TAS2R38 that can be activated by amarogentin [14]. Meanwhile, more effects of bitter compounds become apparent, as the expression of TAS2Rs could be detected in various extra-gustatory organs. The signaling pathway of bitter compounds is dependent on the particular cell type. Deshpande and colleagues showed that activated TAS2Rs expressed on bronchial smooth muscle cells eventually lead to bronchial relaxation [15]. In airway epithelial cells, bitter compounds induce the synthesis of NO, and increase the ciliary beat frequency [16]. In gustatory cells, intracellular calcium influx induces transmitter release [17], and in keratinocytes, ligand binding induces calcium influx and the expression of differentiation proteins [12,13]. In this work, we addressed the question if the TAS2R agonist *Gentiana lutea* extract (GE) may improve the skin barrier by modulating the lipid metabolism in keratinocytes apart from inducing differentiation.

## 2. Results

### 2.1. GE Enhances Lipid Synthesis in Keratinocytes

**Figure 2:**
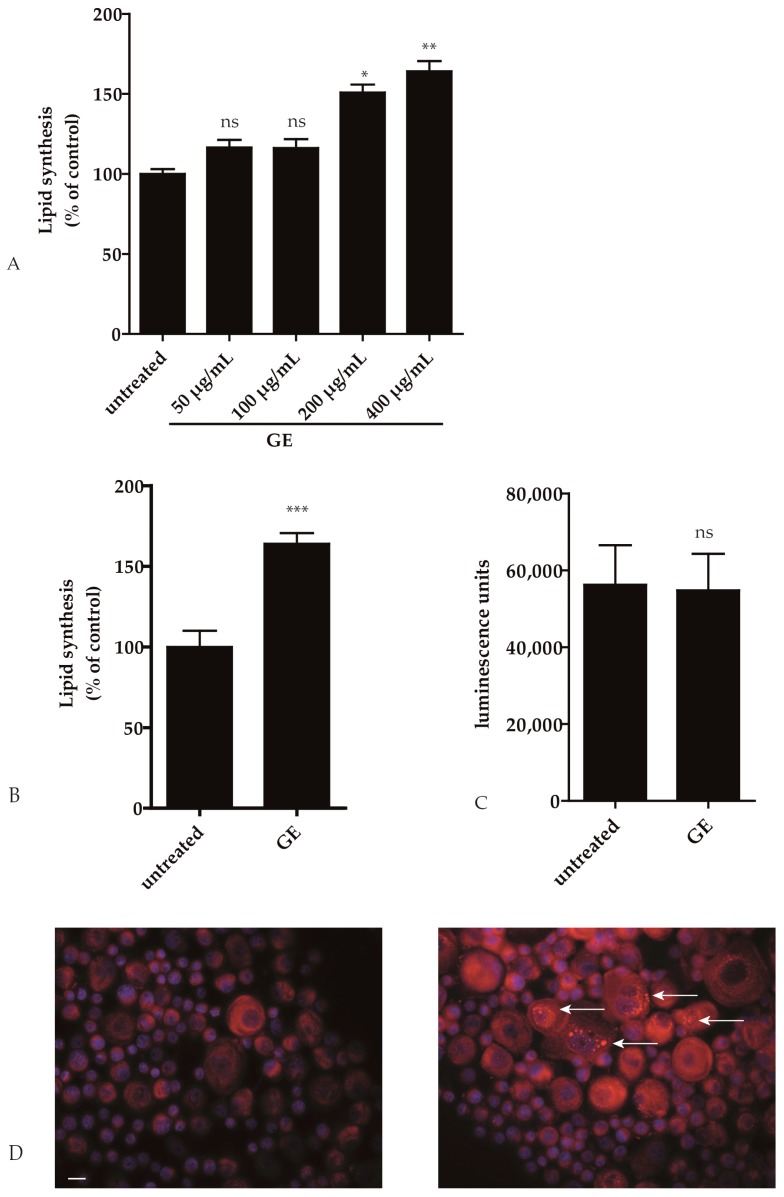
*Gentiana lutea* extract (GE) enhances lipid synthesis in hPK. (**A**–**D**) HPKs were treated with 200 µg/mL GE (unless otherwise stated) for 6 days. The graph in (**A**,**B**) reports the quantitative measurement of intracellular neutral lipids as assessed after Nile Red labeling. Graph C shows cell viability as assessed with the ViaLight Plus bioassay kit; Graph (**D**) shows the qualitative measurement of intracellular neutral lipids after labeled by Nile Red; the nuclei were counterstained with DAPI. The white arrows point to lipid droplets. (ns: not significant, * *p* < 0.05; ** *p* < 0.01, *** *p* < 0.001). The pictures were photographed at a magnification of 400×. The bar indicates 10 µm.

To analyze if GE has an impact on lipid metabolism in keratinocytes, we performed a quantitative fluorescence assay with Nile Red. GE dose dependently enhanced neutral lipid accumulation in HaCaT keratinocytes. At 200 µg/mL there was a statistically significant increase in lipid production of GE-treated compared to sham treated cells (*p* ≤ 0.05; Figure 2A). Next, 6 samples of human primary keratinocytes (hPKs) were incubated with GE (200 µg/mL) for 6 days. In GE treated hPKs, a significantly enhanced neutral lipid accumulation was measured (*p* ≤ 0.001; Figure 2B). No toxic effect of GE could be detected in hPKs as examined with an ATP assay (Figure 2C). The increased number of lipid droplets in hPKs after GE treatment can also be seen in the fluorescence microscope after Nile Red labeling (Figure 2D).

The content of fatty acids (newly synthesized or separated from ceramides) in HaCaT cells and hPKs was determined by gas chromatography with Flame Ionization Detector 96 (GC-FID) and compared with data from the literature. The percentage of fatty acids and the fatty acid profiles of hPKs isolated from biopsies were comparable to cultured keratinocytes (see Table 1).

**Figure 3:**
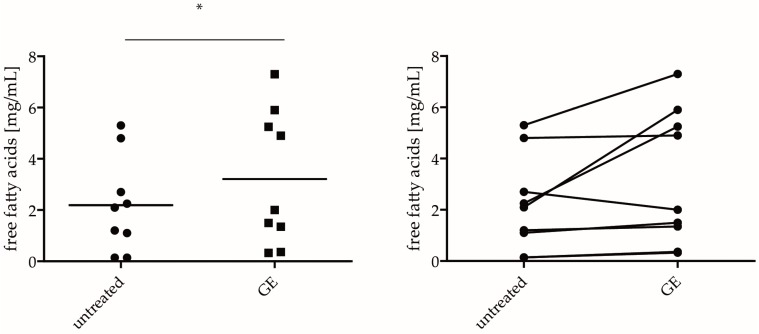
GE increases the amount of fatty acids in vitro. hPKs were treated with 200 µg/mL GE for 6 days, and the lipid fraction was isolated and analyzed by GC-FID. The results were shown in a scatter blot (left graph), and in an untreated versus treated graph that shows the individual treatment effect within cells of the same skin biopsy (right graph). The *p* value is indicated in the figure by an asterisk (* *p* < 0.05).

There were some differences in C16:1 or C18:2 expressions. This might be caused by the cell culture medium. Ponec and colleagues described, for example, that fetal calf serum-containing culture medium contains low levels of C18:2, and high levels of C16:1, apart from individual differences within different hPKs [18]. Nevertheless, the results are comparable and provide a suitable model to study epidermal lipid metabolism. Eight samples of hPKs were incubated with GE (200 µg/mL), and showed a significantly increased amount of fatty acids (*p* < 0.01) (Figure 3). hPKs either responded to GE stimulation, or showed no increase of fatty acids (Figure 3, second graph).

The reason why some hPKs do not respond to GE might be the occurrence of polymorphisms in bitter taste receptors [19].

GE-induced lipid production was seen in both young and old keratinocytes (Supplementary Table S1A,B; Supplementary Figure S1). In particular, the fatty acids palmitic acid (C16:0) and linoleic acid (C18:2) were increased after GE treatment (Supplementary Table S1) and showed an average increase of these fatty acid between 2 -and 4-fold. The absolute values vary between 0.02 and 1.3 mg/mL of these fatty acids.

### 2.2. Pathways Involved in GE-Induced Lipid Production

**Figure 4:**
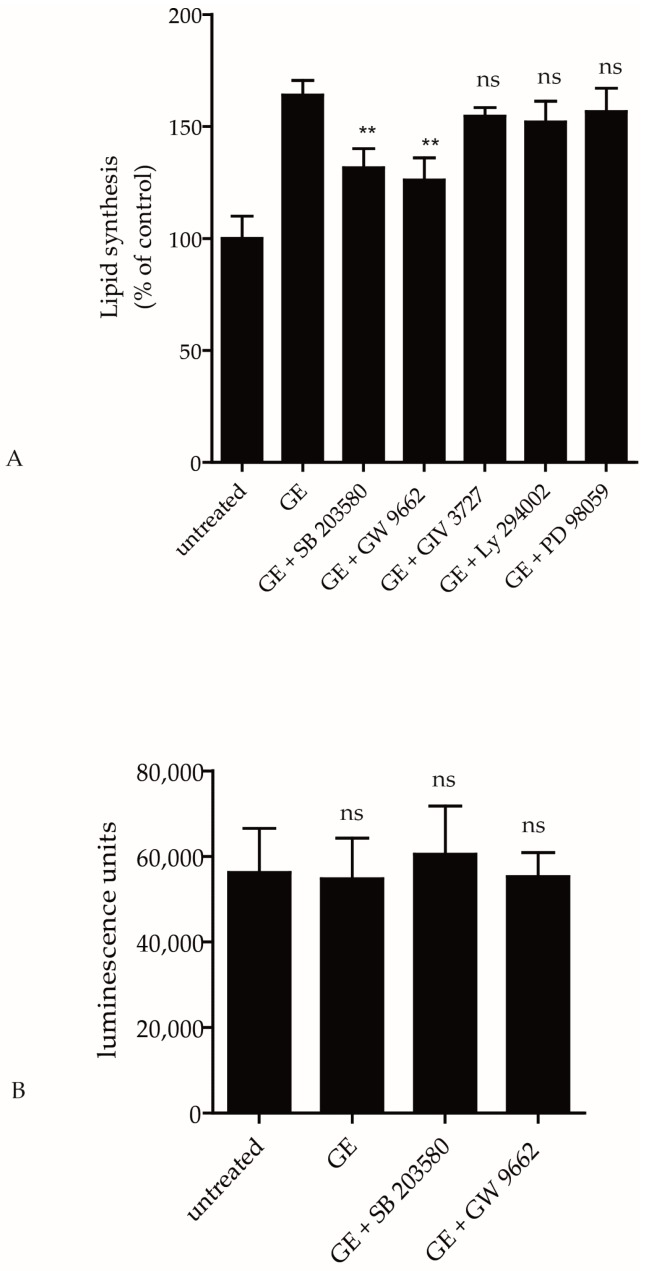
GE-induced lipid synthesis involves the MAPK and PPAR pathway. hPKs were cultured in 96 well plates and were treated with vehicle, GE (200 µg/mL), or with GE and one of the inhibitors (p38 MAPK inhibitor SB203580, PPARγ antagonist GW9962; NFκB inhibitor GIV 3727, phosphatidyl-inositol-3-kinase inhibitor LY294002 or ERK inhibitor PD98059) for 6 days. (**A**) Quantitative measurement of intracellular neutral lipids as assessed after Nile Red labeling and (**B**) measurement of cell viability as was assessed with the ViaLight Plus bioassay kit. All Data are expressed as means ± SD of 6 independent experiments with 8 replicates respectively (ns—not significant, ** *p* < 0.01).

Peroxisome proliferator activated receptors (PPARs) play a crucial role in lipid and glucose homeostasis. Rau and colleagues showed that PPARγ can be activated by GE [20]. Therefore, we tested if a PPARγ antagonist (GW9962) can inhibit the lipid synthesis in hPKs. GE-induced lipid synthesis in hPKs could be antagonized by GW9962 (*p* < 0.05; Figure 4A). To elucidate further signaling pathways possibly involved in GE-induced lipid synthesis, we used several inhibitors that are involved in intracellular signaling in human keratinocytes, e.g., phosphatidyl-inositol-3-kinase (PI3K) inhibitor (LY294002), inhibitors of the MAP-kinase pathway (ERK inhibitor (PD98059) and p38 mitogen-activated protein kinase (MAPK) inhibitor (SB203580)), or NFκB inhibitor (GIV 3727). As seen in Figure 4A, GE-induced lipid synthesis was not modified by the PI3K inhibitor, ERK inhibitor or NFκB inhibitor (Figure 4A). In contrast, the PPARγ inhibitor, as well as the p38 MAPK inhibitor, significantly antagonized the effect of GE-induced lipid production (*p* ≤ 0.01; Figure 4A). These inhibitors showed no cytotoxic effects in hPKs (Figure 4B). This suggests an important role of the MAPK p38 and PPARγ pathway in GE-induced keratinocyte lipid synthesis.

### 2.3. GE Modulates the Activity of Ceramide Metabolic Enzymes

**Figure 5:**
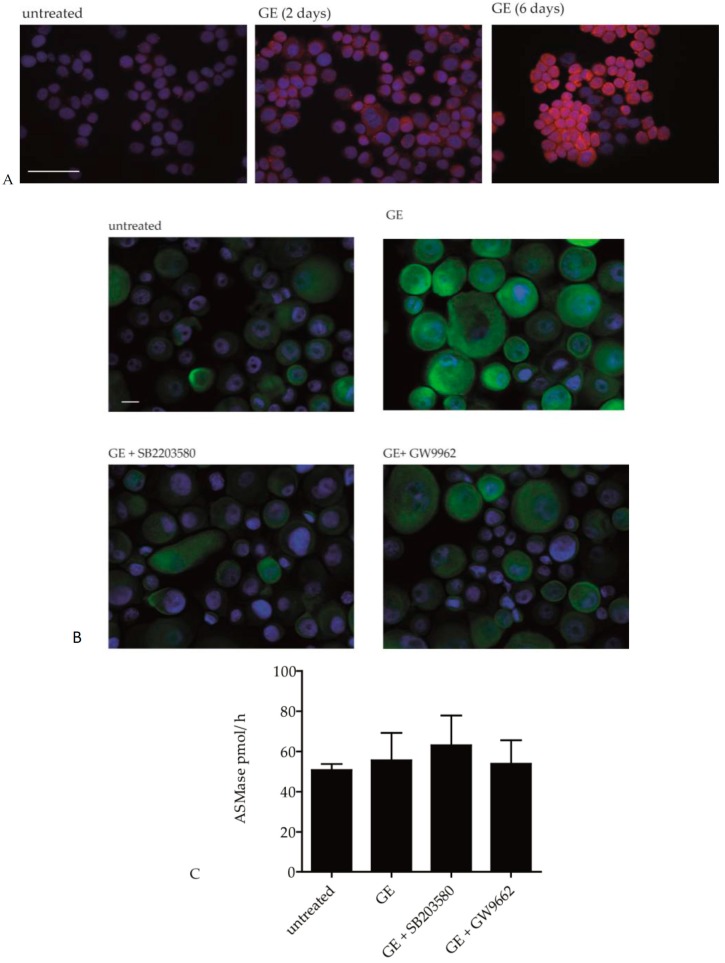
GE induces CerS3 expression but not sphingomyelinase activity. (**A**) hPKs were treated with GE (200 µg/mL) for 0, 2, or 6 days. Then, the cells were stained against CerS3. The pictures were photographed at magnification of 100×, and the bar indicates 50 µm; (**B**) hPKs were treated with GE (200 µg/mL), or with GE and one of the inhibitors (PPARγ antagonist GW9962 or p38 MAPK inhibitor SB203580) for 6 days. Then, the cells were stained against CerS3. The pictures were photographed at a magnification of 400×, and the bar indicates 10 µm; (**C**) hPKs were treated with GE (200 µg/mL) or with GE and inhibitors, as indicated, for 6 days. Then, the sphingomyelinase activity was measured in the cell lysates. Data are expressed as means ± SD of three independent experiments (ns—not significant).

To investigate if GE influences ceramide synthesis, we measured the expression and activity of key enzymes that are involved in ceramide metabolism, e.g., CerS3 and acidic sphingomyelinase. Immunohistochemical staining showed that CerS3 expression was increased in GE-treated HaCaT cells. This effect was more prominent after 6 days of GE treatment compared to 2 days (Figure 5A). To assess if this effect is PPARγ and/or p38 MAPK dependent, hPKs were pre-incubated with SB203580 (p38 MAPK inhibitor) or GW9962 (PPARγ inhibitor) for 1 h before the GE treatment. It turned out that GE-induced CerS3 expression could nearly be reduced to background level, either by blocking the p38 MAPK or PPARγ pathway (Figure 5B). These results indicate that an altered expression of the ceramide metabolic enzyme, CerS3 that is nearly exclusively expressed in hPKs, might account for the increased lipid content in GE-treated hPKs. In contrast, acidic sphingomyelinase activity was not affected by GE treatment (Figure 5C).

### 2.4. GE Enhances Lipid Production In Vivo

**Figure 6:**
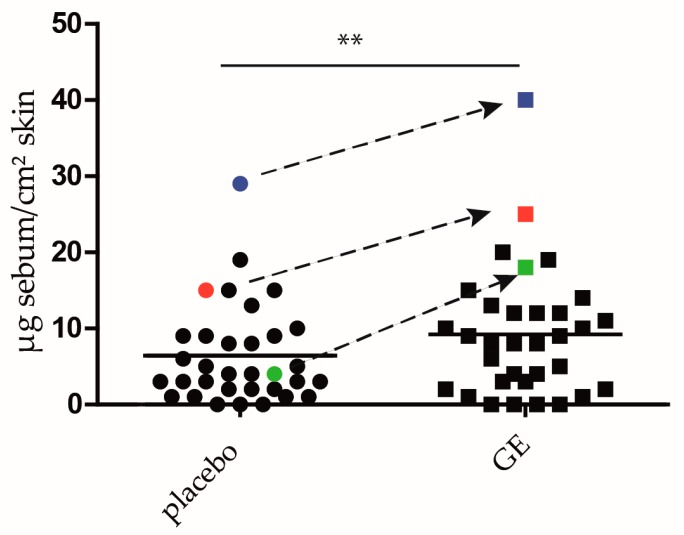
GE increases lipid synthesis in vivo. In a placebo controlled half side comparison, 33 adult volunteers were treated on their volar forearms with 5% GE cream, or placebo cream. The box blots show the lipid content of the skin after 2 weeks. The red, green and blue dots and squares correspond to one volunteer respectively. The arrows show the difference in the lipid content between the placebo and GE-treated forearm. The *p* value is indicated in the figures by asterisks (** *p* < 0.01).

To analyze if GE also increases lipid synthesis in vivo, the effect of a 5% GE cream was assessed in a proof of concept placebo-controlled half side comparison in 33 healthy volunteers. One of their volar forearms was treated with a defined amount of GE cream for 4 weeks, twice a day, whereas the other forearm received the placebo cream. We chose the volar forearm, because, in this body region, skin lipids are nearly exclusively produced by keratinocytes. In other skin regions, the lipids are mainly produced by sebaceous glands. After 2, 3, and 4 weeks, the lipid content was measured using a sebumeter. Already after 2 weeks of treatment, a significant increase in the lipid content could be seen (Figure 6). Thirteen volunteers (39%) were non-responders, because they showed no change in the lipid content. Twenty volunteers (60%) showed an increase in the lipid content of at least 25%, and 8 of them showed an increase of 50% or more. The colored dots in Figure 6 show 3 responders. This effect is sustained throughout the whole study (Supplementary Figure S2).

## 3. Discussion

Skin surface lipids, and bound water in the stratum corneum, contribute to an intact skin barrier. An increase in fatty acids and their metabolites can activate the nuclear transcription factors PPARs, and induce the gene expression of proteins that are required for corneocyte formation (e.g., involucrin) [1]. PPARs also stimulate the formation and packaging of lipids, and can induce ceramide synthesis [21]. Rau and colleagues described that a GE extract activates PPARγ [20]. These findings fit to our results that GE induces lipid synthesis in hPKs, and that this effect can be blocked with a PPARγ inhibitor. PPARγ is a key regulator of lipid metabolism in different cell types (e.g., in macrophages [22], sebocytes [23], and dendritic cells [24]). Another pathway involved in the lipid metabolism of keratinocytes is the MAPK pathway, because GE-induced lipid production could be diminished by a p38 MAPK inhibitor. Inappropriate MAPK signaling contributes to the development of several diseases, for example, the metabolic syndrome [25]. p38 MAPK also influences lipogenesis in adipocytes [26]. This is in contrast to the effect of p38 MAPK in the hepatic metabolism, where it stimulates gluconeogenesis, while simultaneously inhibiting lipogenesis [27]. The MAPK pathway is also involved in endocannabinoid-induced lipid synthesis in sebocytes, as shown by Dobrosi and colleagues [23]. However, epidermal lipids are composed of a mixture of almost equal proportions of ceramides, free fatty acids and cholesterol, whereas sebaceous lipids are non-polar lipids, such as triglycerides, wax esters, and squalene [28,29]. In the present study, we could show that GE induces lipogenesis in hPKs. Bonté and colleagues described already in 1996 that the bitter extract of *Simarouba amara*, a tree that grows in in the rainforest of Brazil, shows a marked increase in total lipids in air-exposed keratinocyte cultures [30]. This bitter compound can possibly induce the lipid synthesis in keratinocytes comparable to GE, although this publication attached no importance to the fact that *Simbarouba amara* extract tastes bitter and might activate bitter taste receptors. Another substance that modulates lipogenesis, and especially, ceramide production in keratinocytes, is vitamin C. However, the activity of sphingomyelinase, a hydrolase enzyme that catalyzes the conversion of sphingomyelin to ceramide, remained unaltered after vitamin C treatment [5]. These observations are in line with our finding that GE increases CerS3 expression in keratinocytes without affecting acidic sphingomyelinase activity. CerS3 is particularly important for skin cornification [31]. Its function cannot be replaced by any of the other five CerS, and ceramide 3 is exclusively expressed in the skin and testis [32]. In general, ceramides are sphingosines (fatty amino alcohols) that are *N*-acylated with various fatty acids to form many ceramide species [33] that are involved in keratinocyte maturation.

In atopic dermatitis and psoriasis, the ceramide concentration in the stratum corneum is diminished [6,34,35]. In particular, the levels of ceramide 1 and 3 are significantly lower compared to healthy people [36]. Vitamin D or vitamin D analogues, such as calcipotriol, are used for the treatment of psoriasis. They increase the ceramide level by inducing sphingomyelin hydrolysis in human keratinocytes [37]. In this context, it is interesting that ceramides also have an anti-proliferative effect [38]. Certain amphiphilic lipids, in particular sphingosines and free fatty acids (e.g., lauric acid), decrease the growth of microbes, and thereby reduce infections [29]. A decreased level of sphingosine is associated with increased *Staphylococcus aureus* colonization in atopic dermatitis patients [39]. Moreover, free fatty acids that are formed by phospholipid breakdown contribute to the acidification of the stratum corneum [40]. This acidic environment is very important as it regulates many enzymes in the stratum corneum, e.g., acidic sphingomyelinase [41].

In a placebo-controlled double-blind half side comparison, we tested the effect of GE on lipid synthesis on the volar forearm. We could demonstrate that GE also increases the lipid content of the stratum corneum in vivo. This could be of therapeutic value in atopic dermatitis that is characterized by an impaired epidermal barrier with reduced lipid content. Similarly, body regions in which lipids are exclusively produced by epidermal cells, for example around the lips, in the hollow of the knee and the bend of the elbow, might benefit from topical GE treatment. Interestingly, these body regions are also particularly affected by atopic dermatitis. It is noteworthy that a ceramide, cholesterol, and fatty acid mixture, corresponding to the epidermal lipid composition, can accelerate the recovery of the skin barrier [42,43].

It is commonly accepted that ceramides are important for skin health. However, studies performed with human fibroblasts that were incubated with a ceramide analogue in an interleukin-6 (IL-6) rich environment, have shown that ceramides also modulate the secretion of prostaglandin E_2_ (PGE_2_) [44]. This indicates that ceramides are not only beneficial for skin barrier recovery, but may also enhance the inflammatory responses to inflammatory stimuli. [36]. Also, it has been shown that ceramides can activate the NLRP3 inflammasome in obesity-induced inflammation [45]. To confirm that GE does not induce inflammatory responses in keratinocytes, we measured PGE_2_ and IL-6 release of hPKs after GE treatment. No expression of these inflammatory mediators could be detected in our study (data not shown). On the other hand, Danso and colleagues showed in skin equivalents that cytokine addition modulates lipid synthesis. As a consequence, inflammation might cause lower ceramide levels that are also observed in atopic dermatitis and psoriasis [46].

In summary, the lipid content of the stratum corneum is essential for an optimal epidermal barrier function. GE enhances epidermal lipid synthesis and might therefore be useful to improve skin disorders with barrier abnormalities, e.g., very dry skin and atopic dermatitis.

## 4. Materials and Methods

### 4.1. Antibodies and Reagents

The polyclonal rabbit-anti-human CerS3 antibody was from Antikörper-online (Aachen, Germany; 1:50). The secondary antibodies were the swine anti-rabbit-FITC antibody (Dako, Hamburg, Germany; 1:30) and the donkey anti-rabbit-Alexa-Fluor 555 antibody (Thermo Fisher Scientific, Darmstadt, Germany). DAPI was from Sigma-Aldrich GmbH (Taufkirchen, Germany). The following specific inhibitors were used: PPARγ antagonist (GW9962, Enzo, Lörrach, Germany), PI3K inhibitor (LY294002; Sigma-Aldrich GmbH), ERK inhibitor (PD98059, New England Biolabs GmbH, Frankfurt, Germany), p38 MAPK inhibitor (SB203580, Sigma-Aldrich GmbH) and NFκB inhibitor (GIV 3727, Merck Millipore, Darmstadt, Germany). Methanolic sodium hydroxide solution, hexane standards, loganic acid, and loganin were from Carl Roth GmbH (Karlsruhe, Germany). Boron trifluoride–methanol complex was from Merck KGaA (Darmstadt, Germany), and standard methyl heptadecanoate was from Sigma-Aldrich GmbH. Orto-phosphoric acid, 85%, Ph. Eur. p.a. grade was supplied by VWR. Amarogentin and gentiopicroside were purchased from Chromadex Inc. (Santa Ana, CA, USA).

### 4.2. Preparation of Yellow Gentian Root High Pressure Ethanol (HPE) Extract

Whole gentian root was purchased from a Bavarian farm specialized in root drugs (Berghof Kräuter GmbH, Heilsbronn, Germany). The root was passed through a cutting mill with 3 mm sieve (Pallmann Maschinenfabrik GmbH, Zweibrücken, Germany). The powder was filled into a high pressure extractor and percolated with 8 kg of 96% (*v*/*v*) ethanol at 110 bar/60 °C per kg feedstock to give the crude HPE (high pressure ethanol) extract. The major ethanol amount was removed by vacuum distillation, in order to adjust the final spissum extract to 50% dry matter, according to a drug/extract ratio of about 2.5/1.

### 4.3. HPLC-DAD Analysis of Yellow Gentian Root HPE Extract

All solvents were of HPLC grade and supplied by VWR International GmbH (Darmstadt, Germany). Standards were dissolved in methanol at 0.3–0.7 mg/mL concentration. The yellow gentian root HPE extract was dissolved under sonication in methanol at 8–10 mg/mL concentration and filtered through a PTFE (polytetrafluoroethylene) filter with 0.45 µm pore diameter before injection.

**Figure 7:**
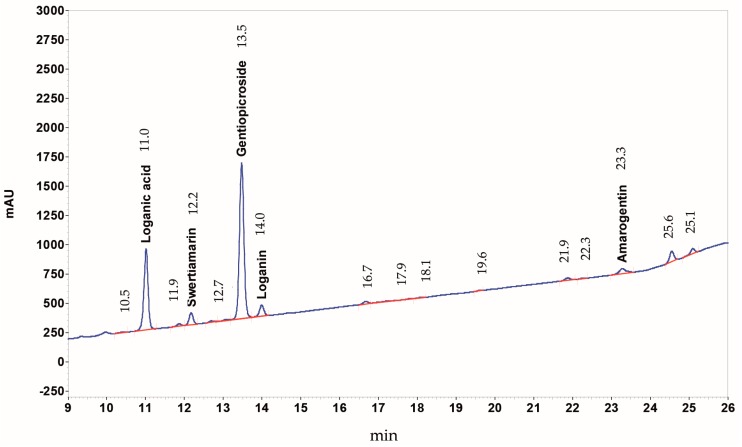
HPLC-UV chromatogram of yellow gentian root CO_2_-extract. The detection was at 232 nm: peak identifications (concentrations) are: loganic acid (3.1%), swertiamarin (0.79%), gentiopicroside (12.3%), loganin (0.41%), and amarogentin (0.05%).

A Merck Hitachi LaChrom Elite HPLC system was used, consisting of auto sampler L-2200, pump L-2130, DAD detector L-2420, column oven L-2350, operated with software EZ Chrom Elite Version 3.3.2 Build 1037 (SP2); column Lichrospher 100 RP-18e (5 µm), 250 × 4 (mm length × internal diameter), Merck KGaA (Darmstadt, Germany). Eluent A was water acidified with *o*-phosphoric acid 85% to pH 2.3–2.6, eluent B acetonitrile, eluent C *1*-propanol; gradient profile: ramp 1: 98% A, 1% B, 1% C to 70% A, 15% B, 15% C within 20 min; ramp 2: to 60% A, 20% B, 20% C within 2 min., isocratic for 10 min. Eluent flow was 1 mL/min, oven temperature 30 °C, detector 232 nm, injection volume 10 µL. The concentration of Swertiamarin is calculated as Gentiopicroside, because this compound was not available as reference standard. Peak identification was based on literature. A HPLC-UV chromatogram of yellow gentian root CO_2_-extract is shown in Figure 7.

### 4.4. Cell Culture

The human keratinocyte cell line HaCaT was from CLS (Cell Lines Service; Heidelberg, Germany) and was cultured in Dulbecco’s modified essential medium (DMEM; Biochrom, Berlin, Germany) containing 10% fetal calf serum (FCS; PAA, Pasching, Austria). hPKs were prepared from juvenile foreskin or adult skin obtained from dermatological surgery, and cultured according to the method of Rheinwald and Green [47] in DermaLife medium (Cell System, Goisdorf, Germany). All cells were cultured at 37 °C in a humidified atmosphere with 5% CO_2_.

### 4.5. Immunofluorescence

hPKs were incubated for 6 days with 200 µg/mL GE and centrifuged on slides. Then, the cells were stained with the polyclonal rabbit anti-human CerS3 antibody. The required secondary antibodies (donkey anti-rabbit-Alexa-Fluor 555 or swine anti-rabbit-FITC) were applied for 1 h at room temperature, according to the manufacturer’s instructions. Then, the cells were stained with DAPI and mounted in fluorescent mounting medium (Dako). Images were taken with a fluorescence microscope (Carl Zeiss, Oberkochen, Germany) equipped with Axiovision software version 4.1.

### 4.6. Cytotoxicity Assay

hPKs were incubated in a 96 well plate (2000 cells per well) and incubated for 6 days with GE (200 µg/mL) before the cytotoxicity was assessed with the ViaLight Plus ATP assay (Lonza, Basel, Switzerland), according to the manufacturer’s instruction. The method is based on the bioluminescent measurement of ATP that is present in metabolically active cells. Luciferase catalyzes the formation of light from ATP and luciferin. The emitted light intensity is directly proportional to the ATP concentration, and is measured with a luminometer (Sirius HT, BioTek, Bad Friedrichshall, Germany).

### 4.7. Enzyme Activity Assay for Sphingomyelinase

hPKs were incubated for 6 days with 200 µg/mL GE. Subsequently, the cells were lysed and processed according to the manufacturer’s protocol (Acid Sphingomyelinase Activity Colorimetric Assay Kit, BioVision, San Francisco, CA, USA). The absorption was measured at 570 nm on an ELISA reader (Sirius HT).

### 4.8. Determination of Intracellular Lipids in Keratinocytes

For semi-quantitative detection of lipids in keratinocytes, hPKs were treated for 6 days with GE (200 µg/mL) and stained with Nile Red (9-diethylamino-5*H*-benzo[a]phenoxazine-5-one, Sigma-Aldrich GmbH). This dye is only strongly fluorescent in a hydrophobic environment, and can also be applied to cells in an aqueous medium, because it does not dissolve lipids [2]. Staining was performed in formalin-fixed cells with 100 ng/mL Nile Red in phosphate buffered saline PBS for 5 min. Then, the cells were counterstained with 4′,6-Diamino-2-phenylindol Dihydrochlorid (DAPI, Sigma-Aldrich GmbH) [2]. For quantitative measurement, hPKs (4000 cells/well) were cultured in 96 white well clear bottom plates in eight replicates, and treated with GE for 6 days according to the literature [10]. For all samples, the average of six experimental settings was used. The supernatants were then discarded, and 100 µL Nile Red solution (1 µg/mL) was added to each well. The emitted fluorescence was measured on an ELISA reader, using 485 nm excitation and 590 nm emission wavelength for neutral lipids [23]. The relative fluorescence units from the untreated control were shown as 100%, and the GE-treated samples related to this value.

### 4.9. Isolation of Lipids from Keratinocytes 

hPKs were incubated with 200 µg/mL GE. The lipid content was measured after 6 days of GE incubation. The age of the hPK donors varied from 2 to 92 years. Four samples were classified as young (<10 years), and 4 as old (>50 years), as shown in the supplementary table to have a representative group. The average age of the young group was 7.3 ± 3.6 years, and of the older group, 71.0 ± 16.8 years.

The lipid extraction was performed as described by Bligh and Dyer [48]. Briefly, the cells were pelleted and re-suspended in a water-chloroform-methanol mixture (4:5:10), sonicated, and centrifuged. Then, the upper layer was mixed with chloroform-water (1:1) and centrifuged. The lower chloroform layer with the lipids was dried in a heating block, and stored at −80 °C until it was used for analysis of fatty acids by GC-FID.

### 4.10. Gas Chromatography with Flame Ionization Detector (GC-FID) Analysis of Fatty Acids

**Figure 8:**
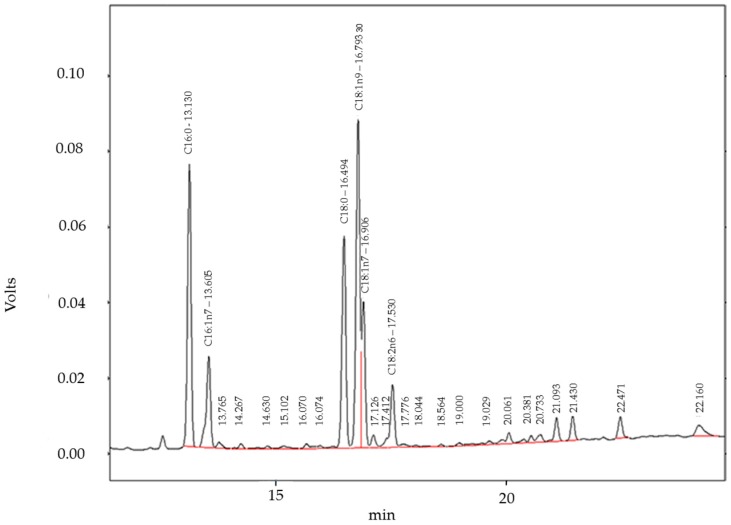
The graph shows a representative GC-FID chromatogram of fatty acids GC-FID chromatogram of fatty acids extracted from the cell lipids. The first two peaks between 13 and 14 min retention time are C16:0 and C16:1n7 acids. The second group between 16 and 17 min are C18:0, C18:1n9, and C18:1n7 (shoulder) acids, and the smaller peak at 17.5 min is C18:2n6 acid. The red line indicates the base for peak integration.

All solvents and reagents were of analytical grade. Fatty acids were analyzed according to the DGF standard method C-VI 10a (00) by transmethylation with boron trifluoride (BF3). Cell samples were saponified with 200 µL methanolic sodium hydroxide solution, 0.5 mol/L. Resulting soaps were converted to methyl esters by addition of 100 µL of a boron trifluoride–methanol complex, 20% solution. Reaction time of each step was 10 min, and carried out in a water bath at 80 °C. The fatty acid methyl esters were diluted in 400 µL hexane and centrifuged. The clear hexane layer was transferred into a vial and used for GC analysis. A Shimadzu GC 17 A with class VP 4.3 software (Shimadzu Europe GmbH, Duisburg, Germany) equipped with AOC20i autosampler and a Roticap FFAP column (Carl Roth GmbH, 30 m × 0.25 mm i.d., 0.25 µm film thickness) was used. Carrier gas was nitrogen, with constant flow of 1.6 mL/min; initial GC oven temperature was 110 °C, increased to 220 °C at a rate of 5 °C/min and held at 220 °C for 20 min; injector temperature was 240 °C, injection volume was 8 µL with split mode 1:5; FID temperature was 280 °C. The absolute content of each fatty acid (newly synthesized or separated from ceramides) in hPKs, and the relative fatty acid composition, were identified by comparing the retention time of samples with the lipid standard “Fatty Acid Methyl Ester Mix” (Sigma-Aldrich GmbH). Quantification of fatty acids was done with external standard methyl heptadecanoate, purity >99% (Sigma-Aldrich) at a concentration of 1 mg/mL. The accuracy was determined by the recovery method to be 98%; repeatability tests showed relative standard deviation (RDS) of less than 4% for all fatty acid methyl esters. A representative GC-FID chromatogram of fatty acids extracted from the cell lipids is shown in Figure 8.

### 4.11. Half Side Comparison

In a half side comparison, 33 adult volunteers with normal to dry skin were treated with 5% GE cream on one of the volar forearms for 4 weeks. The other arm was treated with the vehicle unguentum emulsificans aquosum (INCI: nonionic emulsifying alcohols, 2-ethylhexyllaurate, glycerol, potassium sorbate, aqua dest.). The volar forearms were chosen, because, at this area, the lipids are mainly produced by keratinocytes, and not by sebaceous glands, as in other regions of the skin. The study protocol (code 2013-VE01) was approved by the ethics committee of the University of Freiburg, and written informed consent was obtained from all subjects. The approval number is 11/12. Included were subjects of both sexes (4 men and 29 women), with an average age of 43.6 ± 13.2 years. Exclusion criteria were the usage of cosmetics or topical therapeutics in the test area. The verum or placebo cream was applied in a double blind manner to the test area by the study participants, two times daily for 4 weeks. Study visits were at day 1 (U1), after 2 weeks (U2), after 3 weeks (U3) and after 4 weeks (U4). At each visit, the total skin surface lipids (sebum and corneal lipids) were measured in the test area using a sebumeter SM 810 (Courage + khazaka electronic GmbH, Köln, Germany). The mat tape of the sebumeter is brought into contact with skin and becomes transparent in relation to the sebum on the test area. Then, the transparency of the tape is measured by a photocell. The light transmission represents the lipid content. Data are shown in plots. The readings were tested for significant differences between U1 and U2, U1 and U3, as well as U1 and U4, respectively, using the Wilcoxon test for pairwise comparisons. The *p* value is indicated in the figure by asterisks (** *p* ≤ 0.01). All measured values were expressed as the median of 3 adjacent recordings, to avoid measuring inaccuracies.

### 4.12. Statistical Analysis

The in vitro data were analyzed using the unpaired Student *t*-test (two-tailed). The in vivo study was evaluated by Wilcoxon’s signed rank test, and statistical significance was established at * *p* ≤ 0.05 and ** *p* ≤ 0.01. The statistical analysis was performed with the GraphPad Prism software (GraphPad Software, version 5.03, Inc., San Diego, CA, USA). Data are expressed as mean ± SD of at least three independent experiments. In the in vivo half side comparison, three adjacent body regions at the volar forearm were measured.

## Figures and Tables

**Table 1:** The composition of fatty acids isolated from hPKs or the keratinocyte cell line HaCaT, as well as from isolated human epidermis keratinocytes and skin models is indicated as percentage. Own GC-FID data were compared with the data from the literature.

Fatty Acid	% Fatty Acids in hPKs (This Study)	% Fatty Acids in HaCaT Cells (This Study)	% Fatty Acids in Freshly Isolated Keratinocytes of Human Epidermis [18]	% Fatty Acids in Skin Models [18]
C16:0	15.5%	10.8%	15.8%	22.2%
C16:1 w7	4.5%	0.83%	1.8%	13.1%
C18:0	10%	7.8%	14.8%	10.8%
C18:1 w9	11.8%	10%	16.6% (only marked as18:1)	45.8%
C18:2 w6	11.8%	17.5%	23.3% (only marked as C18:2)	1.1%

## Supplementary Materials

Supplementary materials can be found at www.mdpi.com/1422-0067/18/8/1814/s1.

## Abbreviations

ASMase	Shingomyelinase activity
BAK	Bcl-2 antagonist/killer1
Ca^2+^	Calcium
Bcl-2	B-cell lymphoma 2
CerS3	Ceramid synthase 3
GC-FID	Gaschromatography with flame ionization detector
INCI	International nomenclature of cosmetic ingredients
IL-6	Interleukin-6
HPE	High pressure ethanol
HPLC	High performance liquid chromatography
hPKs	Human primary keratinocytes
MDM2	Mouse double minute 2 homolog
p38 MAPK	p38 mitogen-activated protein kinases
PGE_2_	Prostaglandin E2
PPARγ	Peroxisome proliferator activated receptor γ
