# Supplementary material for: The Herbal Bitter Drug Gentiana lutea Modulates Lipid Synthesis in Human Keratinocytes In Vitro and In Vivo
Source: Int J Mol Sci. 2017 Aug 22;18(8):1814. doi: 10.3390/ijms18081814 (PMC5578200; doi:10.3390/ijms18081814)
Supplement: Supplementary file 1 [file ijms-18-01814-s001.pdf]

Table S1A

Raw data: Increase in the fatty acid content in old hPKs

Fatty acid	proband *1924 untreated	proband *1924 GE	proband *1934 untreated	proband *1934 GE	proband *1956 untreated	proband *1956 GE	proband *1966 untreated	proband *1966 GE
C16: 0	0.50 mg/ml 18.8%	1.36 µg/ml 25.8%	0.18 16.8 %	0.25 16.9 %	0.34 16.1 %	1.27 21.4 %	0.9 mg/ml 19.08%	0.84 mg/ml 20.12 %
C16:1 w7	0.29 mg/ml 10.7 %	0.54 mg/ml 10.3%	0.03 2.4 %	0.04 2.9 %	0.17 7.9 %	0.71 11.9 %	0.62 mg/ml 12.63%	0.54 mg/ml 12.9 %
C18: 0	0.27 mg/ml 10.0 %	0.60 mg/ml 11.6 %	0.16 15.3 %	0.25 16.9 %	0.32 15.1 %	0.82 13.7 %	0.53 mg/ml 10.76 %	0.41 mg/ml 9.7 %
C18: 1 w9	0.49 mg/ml 18.1 %	1.0 mg/ml 19.2 %	0.05 4.5 %	0.11 7.2 %	0.30 14.4 %	1.70 28.5 %	1.11 mg/ml 22.7%	1.1 mg/ml 25.8 %
C18: 1 w7	0.20 mg/ml 6.9 %	0.33 mg/ml 6.3 %	0.02 1.9 %	0.03 2.0 %	0.13 6.1 %	0.59 9.9 %	0.4 mg/ml 8.07 %	0.4 mg/ml 10.5 %
C18: 2 w6	0.03 mg/ml 0.9 %	0.29 mg/ml 5.5%	0.03 21.5 %	0.35 24.0 %	0.27 12.8 %	0.11 1.9 %	0.04 mg/ml 0.7 %	0.1 mg/ml 2.8 %
C20: 3 w6 unknown 1	0.04 mg/ml 1.49 %	0.13 mg/ml 2.5 %	0.014 1.4 %	0.01 0.46 %	0.04 1.9 %	0.04 0.60 %	0.12 mg/ml 2.5%	0.08 mg/ml 1.9 %
Total fatty acid	2.25 mg/ml	5.25 mg/ml	1.1 mg/ml	1.5 mg/ml	2.1 mg/ml	5.9 mg/ml	4.8 mg/ml	4.9 mg/ml

Table S1B

Raw data: Increase in the fatty acid content in young hPKs

Fatty acid	proband *2010 untreated	proband *2010 GE	proband *2006 untreated	proband *2006 GE	proband *2005 untreated	proband *2005 GE	proband *2014 untreated	proband *2014 GE
C16: 0	0.46 mg/ml 8.9 %	1.24 mg/ml 17.0 %	0.02 15.4 %	0.07 21.1 %	0.21 mg/ml 17.2 %	0.27 20.0 %	0.415 15.3 %	0.45 mg/ml 22.0 %
C16:1 w7	0.43 mg/ml 8.2 %	0.74 mg/ml 10.2 %	0.01 5.1 %	0.02 6.7 %	0.12 mg/ml 10.3 %	0.11 7.9 %	0.25 9.2 %	1.52 7.4 %
C18: 0	0.30 mg/ml 4.8 %	0.63 mg/ml 8.7%	0.03 21.5 %	0.07 21.3 %	0.27 22.1 %	0.21 15.8 %	0.49 17.9 %	0.398 19.4 %
C18: 1 w9	0.90 mg/ml 17.07 %	2.18 mg/ml 30 %	0.02 14.0 %	0.06 17.1 %	0.26 21.9 %	0.32 23.9 %	0.50 22.2 %	0.321 15.6 %
C18: 1 w7	0.56 mg/ml 10.6 %	1.24 mg/ml 17.0 %	0.01 6.2 %	0.03 9.3 %	0.13 10.7 %	0.13 9.6 %	0.36 13.1 %	0.17 8.2 %
C18: 2 w6	0.06 mg/ml 1.2 %	0.13 mg/ml 1.7 µg/ml	0.01 3.4 %	0.01 4.4 %	0.012 1.0 %	0.06 4.7 %	0.092 3.4 %	0.19 9.2 %
C20: 3 w6 unknown 1	0.06 mg/ml 1.1 %	0.08 mg/ml 1.1 %	0.004 2.7 %	0.007 2.1 %	0.03 2.7 %	0.03 1.8 %	0.065 2.4 %	0.03 1.4 %
Total fatty acid	5.3 mg/ml	7.3 mg/ml	0.14 mg/ml	0.33 mg/ml	1.20 mg/ml	1.35 mg/ml	2.7 mg/ml	2.0 mg/ml

Figure S1

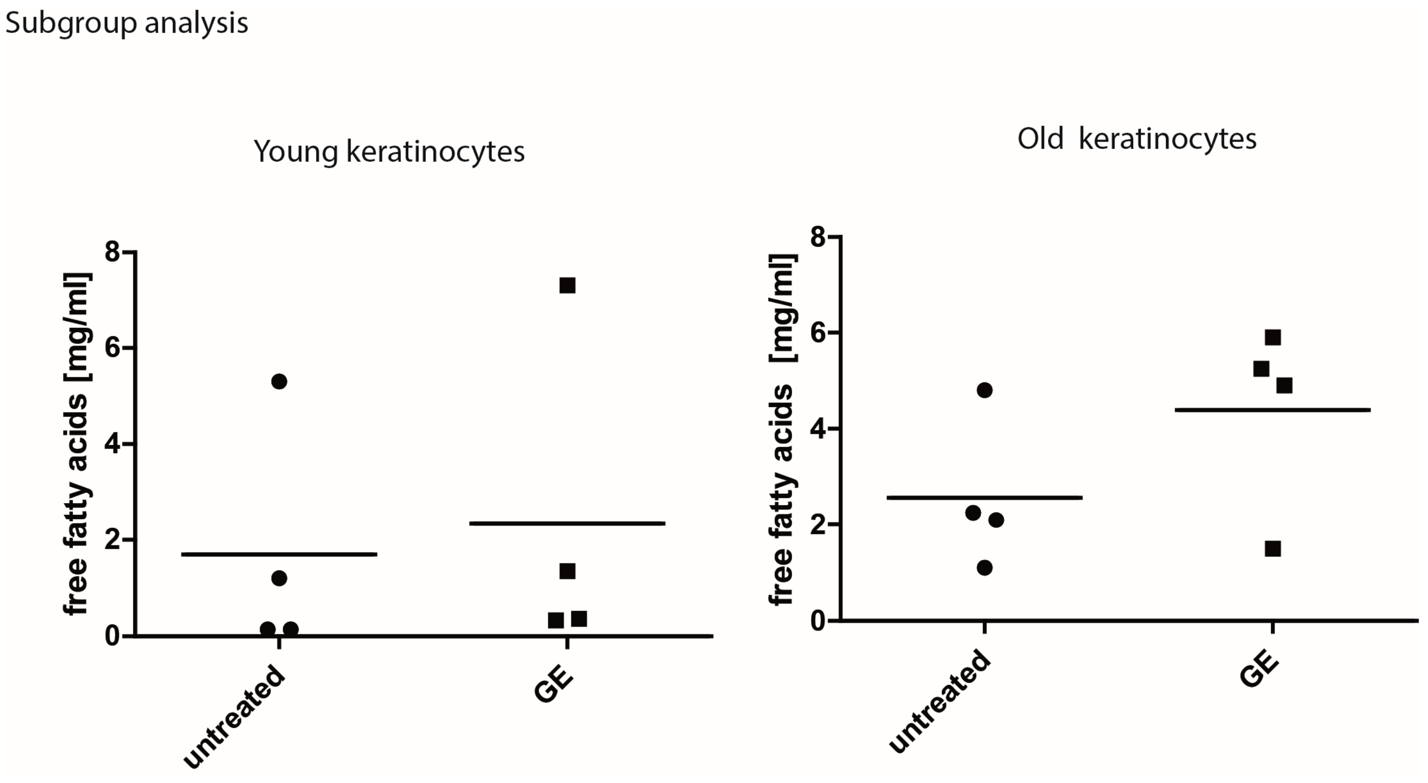

GE increases the amount of fatty acids *in vitro*. HPKs were treated with 200 µg/mL GE for 6 days and the lipid fraction was isolated and analyzed by GC-FID. The results were shown in a scatter blot of keratinocytes from either young or old donors.

Figure S2

A

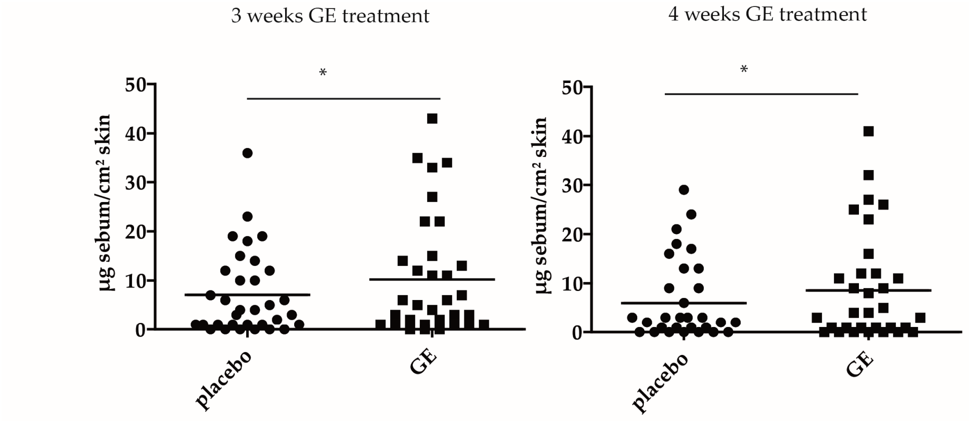

B

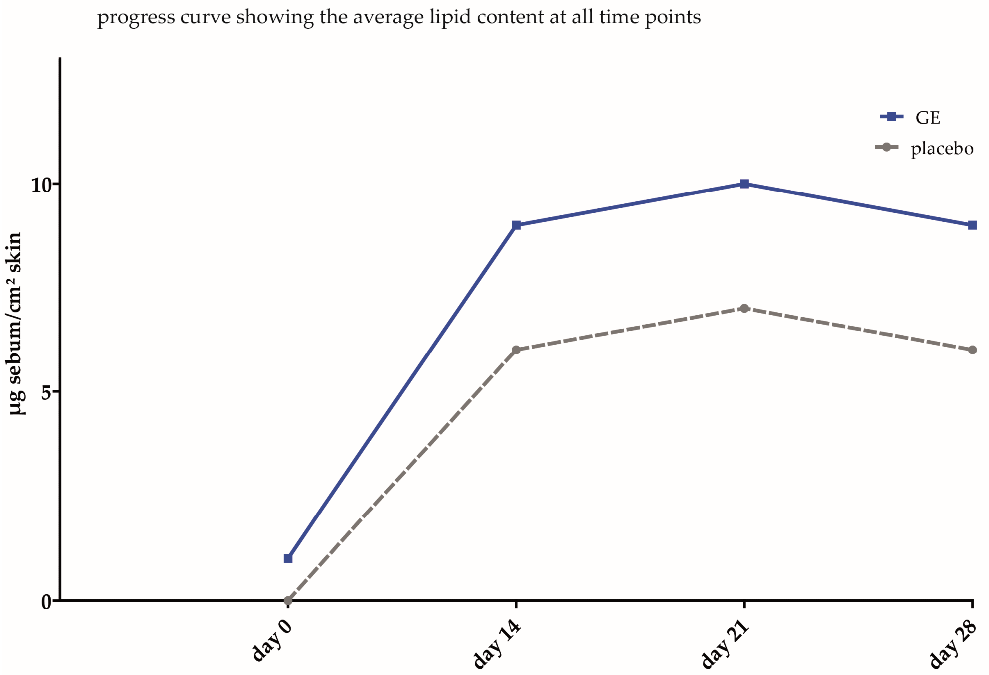

GE increases lipid synthesis *in vivo*. In a placebo controlled half side comparison 33 adult volunteers were treated on their volar forearms with 5 % GE cream or placebo cream. (A) The box blots show the lipid content of the skin after 3 or 4 weeks of treatment. (B) The progress curve shows the lipid content at all time points. The P values are indicated in the figures by asterisks (*P<0.05; **P<0.01).
